# Socioecological factors influencing the risk of developing hypertensive disorders of pregnancy in India: a rapid review

**DOI:** 10.1186/s12884-024-06879-0

**Published:** 2024-10-12

**Authors:** Anumita Alur, Jennifer E. Phipps, Leigh Ann Simmons

**Affiliations:** 1 Health Equity Across the Lifespan Lab, University of California, Davis, CA USA; 2 Betty Irene Moore School of Nursing, University of California, 2570 48th St, Sacramento, Davis, CA 95817 USA

**Keywords:** Hypertensive disorders of pregnancy, India, Bronfenbrenner’s ecological model, Rapid review

## Abstract

**Background:**

The prevalence of hypertensive disorders of pregnancy (HDPs) in India is 11%, which is one of the highest rates globally. Existing research on HDPs in India primarily focuses on biological risk factors, with minimal research on how socioecological factors combine to increase risk of HDPs. We conducted a rapid review using Bronfenbrenner’s Ecological Model to understand the social and cultural factors associated with HDPs among Indian pregnant women to identify possible intervention targets that may uniquely improve health in this population. Bronfenbrenner’s Ecological Model is a framework that can be used to understand the complex relationship between multiple influences on health.

**Methods:**

We reviewed studies published between January 2010 and January 2024 using PubMed, Science Direct, and Scopus databases. Search terms included variants of hypertension, pregnancy, and India. Inclusion criteria were: (1) peer-reviewed journal article; (2) published between January 2010 to January 2024; (3) participants consisted of Indian women living in India; (4) studies evaluated socioecological risk factors associated with HDPs. One independent reviewer performed searches, screening, data extraction, and quality assessment. Each included study was then organized within Bronfenbrenner’s Ecological Model.

**Results:**

A total of 921 studies were generated from the initial search, with 157 exclusions due to duplicates. Following screening for inclusion and exclusion criteria at the title/abstract and full text levels, 17 studies remained in the final review. Socioecological risk factors of HDPs were identified at each level, with the most commonly identified influences including: low socioeconomic status (SES), lacking community education and knowledge on HDP management and prevention, and lacking prenatal HDP screening.

**Conclusion:**

This study determined that the high risk for HDPs in India is influenced by many intertwined socioecological factors. Women in rural and low SES areas need more health education on HDP management and prevention. There also needs to be more adequate prenatal HDP screening, with at least 4 and ideally 8 prenatal visits. Prenatal screenings should be accompanied with culturally appropriate patient education, especially for low SES women who have limited literacy, so that they can effectively make individual and microsystemic lifestyle decisions aimed at either managing or preventing HDPs.

**Supplementary Information:**

The online version contains supplementary material available at 10.1186/s12884-024-06879-0.

## Background

Hypertensive disorders of pregnancy (HDPs) significantly contribute to maternal mortality rates worldwide and include preeclampsia and eclampsia, gestational hypertension, and chronic preexisting hypertension [1]. In India, the prevalence of HDPs is 11%, which remains one of the highest prevalence rates globally [2]. Additionally, South Asians account for 25% of the world’s population, yet carry 60% of the global burden of heart disease (Farrukh et al., 2022). Most maternal mortality due to HDPs occurs in low- or middle-income countries, particularly South Asia and Sub-Saharan Africa [3]. Although these countries are low-resource settings with limited adequate healthcare, most of these global maternal deaths are preventable [2]. Additionally, the prevalence of chronic hypertension in non-pregnant women aged 15–49 in India is already 11.3% [4]. Having HDPs can lead to chronic hypertension or cardiovascular disease (CVD), which further increases this high prevalence of chronic hypertension in women [5]. The information this paper provides can not only be used to reduce rates of maternal mortality from HDPs, but also the risk for chronic hypertension and CVD.

It is important to obtain a holistic view of how HDPs develop to effectively implement prevention and intervention strategies. Currently, we know that the development of HDPs is impacted by a variety of factors, such as genetic markers, family history, diet, exercise habits, substance use, education, and culture [6]. We also know that India has a unique sociocultural milieu, with regions vastly differing in socioeconomic status (SES), food, and culture. In a systematic review by Dhinwa et al. (2021), risk factors and prevalence of HDPs in India were examined [2]. They found that HDP prevalence was highest in the North and lowest in the East. The authors suggested that the difference is due to regional variations in public hospital utilization, lifestyle, diet, and physical activity. Also highlighted in this review is that access and use of prenatal care is not consistent across India and in fact only 21% of pregnant women in India use prenatal services, with socially disadvantaged groups using them far less. Without prenatal care, there are fewer opportunities for early intervention, detection and monitoring of symptoms and risk factors for HDPs, which increases risk. There is a gap in knowledge regarding how these modifiable risk factors combine to impact the development of HDPs in pregnant Indian women. We use the term “women” to inclusively refer to individuals capable of pregnancy.

The purpose of this paper is to answer the following question: How do various socioecological factors interact to impact the risk of developing HDPs in Indian women? This was accomplished by using Bronfenbrenner’s Ecological Model, which is a theoretical framework first described in 1979 that analyzes how the environment shapes our health behaviors and outcomes [7]. This framework proposes that health outcomes are influenced by individual characteristics (i.e., family history, age, education, etc.), one’s immediate environment (i.e. microsystem), the connections between one’s immediate environment (i.e., mesosystem), one’s community and social structures (i.e., exosystem), ideologies and attitudes of the culture (i.e., macrosystem), and environmental changes that occur over the lifecourse (i.e., chronosystem) [7]. This model was chosen because it demonstrates the interdisciplinary nature of how environmental factors in different systems can impact the risk of developing HDPs. The findings at each level of the model may be useful to develop culturally competent programs and policy recommendations for Indian women with HDPs to decrease the incidence rate of hypertension, and ultimately decrease maternal mortality.

## Methods

There is minimal existing research on this topic, and thus a rapid review of the literature was conducted to summarize findings in a timely fashion to guide health policy [8]. While rapid reviews do not currently have a standardized reporting method, we followed a protocol designed based on guidelines outlined by the World Health Organization (WHO) [9] and the Preferred Reporting Items for Systematic Reviews and Meta-Analyses (PRISMA) checklist [10].

### Search strategy

We searched PubMed, Science Direct, and Scopus databases with keywords focused on hypertension (i.e., hypertension, high blood pressure, hypertensive, preeclampsia), pregnancy (i.e., pregnancy, pregnant, birthing people), Indian (i.e., Indian, South Asian), and risk factors (i.e., risk factors, factors, socio-ecological factors, lifestyle factors). Refer to Supplemental Table 1 for the full search strategy. Studies returned from this search in each database were uploaded into Covidence systematic review software (Veritas Health Innovation, Melbourne, Australia. Available at www.covidence.org.) Duplicates were omitted by Covidence, and the remaining studies were manually screened at the title and abstract level against the inclusion and exclusion criteria. Finally, a full text review of eligible studies was conducted.

### Inclusion and exclusion criteria

Studies were selected if they met the following inclusion criteria: (1) published in a peer-reviewed journal; (2) published between January 2010 to January 2024 to provide a current view of the topic in a resource-efficient and timely manner; (3) participants consisted of pregnant Indian women living in India; and (4) evaluated socioecological risk factors associated with HDPs. Studies were excluded if they did not report on hypertension in pregnancy or if they only focused on the biological or genetic aspects of risk for developing HDPs because these are not modifiable factors.

### Data extraction and quality assessment

Data extraction was conducted in Microsoft Excel. Elements extracted from each paper included: study design, objective and outcomes, population, location, and Bronfenbrenner’s Ecological Framework data at each level. Quality assessment was also performed in Microsoft Excel using the Joanna Briggs Institute (JBI) Critical Appraisal Checklist for Systematic Reviews and Research Syntheses [11] or the Mixed Methods Appraisal Tool (MMAT) [12], depending on study design. For each study, we created a percentage to summarize the proportion of items in the assessment that were satisfied.

## Results

### Included studies

**Fig. 1:**
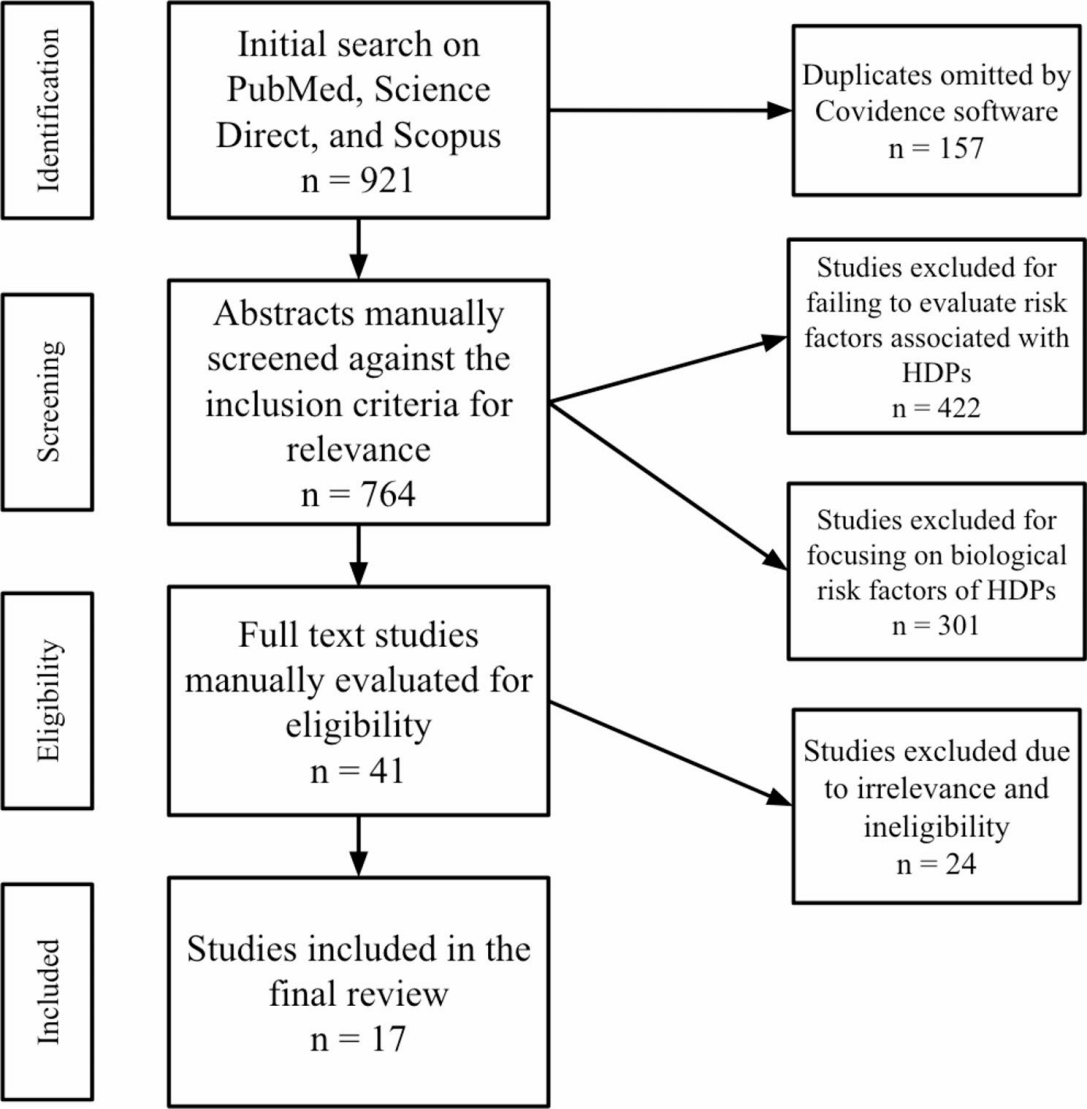
PRISMA flowchart of rapid review process

A total of 921 studies were returned from our searches, 157 duplicates were omitted. The remaining 764 studies were screened at the title/abstract level, which resulted in 723 exclusions. The remaining 41 full-text studies were screened for relevance and eligibility. The majority of articles that were excluded during the abstract screening process did not evaluate risk factors associated with HDPs (*n* = 422). Studies were also excluded because they looked at only the genetic and biological risk factors of the development of HDPs, rather than the socioecological factors (*n* = 301). After this process, 17 studies were included in the final review [2, 6, 13–27]. (see Fig. 1). Each level of Bronfenbrenner’s Ecological Model was represented in the reviewed papers except for the chronosystem level, which refers to how experiences over the lifetime of pregnant women in India may impact their risk for HDPs.

### Data extraction and quality analysis

The study characteristics and major outcomes for each included study are presented in Table 1. Overall, the included studies met recommended quality criteria 94% of the time, 81% for reviews and 97% for qualitative and quantitative studies. See Table 1 for summary quality assessment data and Supplemental Tables 2 and 3 for complete quality assessment tables for reviews and qualitative and quantitative studies, respectively.

**Table 1:** Summary of included studies and quality assessment data

Citation	Title	Objective	Methods	Location	Population	Outcomes	Quality (%)
Agrawal & Fledderjohann (2016) [13]	Hypertensive Disorders of Pregnancy and Risk of Diabetes in Indian Women: A Cross-sectional Study	Investigate the association between preeclampsia and diabetes	Cross-sectional study; A quantitative survey was administered evaluating dietary intake, body mass index (BMI), tobacco-use, alcohol-use, TV watching, and sociodemographic characteristics.	India	Indian women aged 15–49 years old.	HDPs are strongly associated with diabetes in Indian women with risk factors including: tobacco-use, SES, education level, family history of hypertension, and public hospital utilization.	100%
Dhinwa et al. (2021) [2]	Prevalence of Hypertensive Disorders of Pregnancy in India: A Systematic Review and Meta-analysis	Estimate the pooled-prevalence of pregnancy-induced hypertension in India.	Systematic Review; Quantitative pooled prevalence was estimated using mixed-effects and random-effects models.	India	92,220 pregnant Indian women from 18 studies.	Overall pooled prevalence of pregnancy-induced hypertension was 9.09%, indicating the need for implementation of early screening of HDPs.	100%
Farrukh et al. (2022) [6]	Hypertension in Women: A South Asian Perspective	Understand how hypertension development in women differs from men.	Narrative Review; Quantitative and qualitative data was gathered from numerous databases.	India	Does not specify.	There are sex specific factors & environmental differences, like minimal community education on HDPs, associated with hypertension development in women.	72%
Grover et al. (2023) [14]	Hypertension and its Correlates Among Pregnant Women Consuming Tobacco in India: Findings from the National Family health Survey-4	Understand how tobacco-use during pregnancy can impact HDP development in pregnant women.	Cross-sectional study; Quantitative data was obtained from the National Family Health Survey	India (29 states and 7 union territories)	32,428 pregnant Indian women aged 15–49.	There was a higher prevalence of HDP cases amongst those who used tobacco, with SES affecting tobacco-use.	100%
Mathew et al. (2023) [15]	Prevalence of Hypertensive Disorders of Pregnancy, Associated Factors and Pregnancy Complications in a Primigravida Population	Understand the associated risk factors of HDPs and pregnancy complications in primiparous women.	Retrospective analytical study; From the patients’ medical records, quantitative sociodemographic variables were analyzed.	Kerala, India	807 Indian primigravidae of all ages who gave birth at the Pushpagiri Institute of Medical Science & Research Center.	HDPs were found in 18.6% of the population with key risk factors including: age < 18 years and > 40 years, family history of HDPs, and high BMI.	100%
Mehta et al. (2015) [16]	Hypertension in Pregnancy: A Community-Based Study	Study the prevalence and risk factors of HDPs in the rural block of Haryana, India.	Cross-sectional study; Pregnant women registered at 20 community health centers were interviewed to collect quantitative data.	Haryana, India	931 pregnant Indian women registered at 20 community health centers in the rural block of Haryana.	The prevalence of HDPs was 6.9% in this study with major risk factors including: being > 25 years, history of HDP, and history of family hypertension. Authors urged a comprehensive prenatal screening process needs to be implemented in Haryana.	100%
Nath et al. (2021) [17]	Prevalence of Hypertension in Pregnancy and its Associated Factors Among Women Attending Antenatal Clinics in Bengaluru	Assess the prevalence and associated risk factors of HDPs in pregnant women attending the antenatal clinic at three public hospitals in Bengaluru.	Cohort study; A quantitative survey was administered that included: sociodemographic data, social support, domestic violence, marital discord, medical history, obstetric history, and measures of depression and anxiety. Blood pressure was also measured.	Bengaluru, India	783 pregnant Indian women attending the antenatal clinic at three public sector hospitals in Bengaluru.	The prevalence of HDPs was 13.9% in this study with associated risk factors including: high maternal age, low social support, low SES, anxiety, and prenantal depression.	100%
Padhan et al. (2023) [18]	Risk Factors of Preeclampsia: A Hospital-Based Case-Control Study	Determine the risk factors of preeclampsia in Odisha.	Unmatched case-control study; A quantitative survey was administered that included: sociodemographic factors, medical history, and gestational factors. An interview was later conducted.	Odisha, India	100 cases of preeclampsia and 100 controls from the labor room of Veer Surendra Sai Institute were randomly selected.	Family history of hypertension, history of chronic hypertension, and AB blood group were found to be significant risk factors for preeclampsia.	100%
Pal et al. (2017) [19]	Diagnosis, Management and Care of Hypertensive Disorders of Pregnancy (HDP) in India – An Indian Expert Opinion	Evaluate available evidence on this topic and create a treatment plan for screening, diagnosis, care, and management in India.	Narrative Review; Major databases were searched to find quantitative data. A panel of 14 Obstetrics and Gynecology experts were formed.	Mumbai, India	Does not specify.	A need for dietary counseling to pregnant women, and > 4 prenatal visits & initial hypertension screening at 8–12 weeks of gestation.	72%
Panda et al. (2021) [20]	Maternal and Perinatal Outcomes in Hypertensive Disorders of Pregnancy and Factors Influencing It: A Prospective Hospital-Based Study in Northeast India	Identify incidence and risk factors of HDPs to develop appropriate interventions.	Prospective cross-sectional study; Quantitative data was collected and entered in Microsoft Excel.	Northeast India	5460 pregnant Indian women with HDPs at a teaching hospital in Northeast India.	7.4% cases had HDP, 27.6% had gestational hypertension, 27.6% had mild preeclampsia, 33.6% had severe preeclampsia, and 11.2% had eclampsia. 13.4% went to intensive care unit and 2.9% ended in maternal deaths. Routine prenatal screening for HDP is necessary.	85%
Prasad et al. (2021) [21]	Performance of Fetal Medicine Foundation Algorithm for First Trimester Preeclampsia Screening in an Indigenous South Asian Population	Evaluate the performance of the Fetal Medicine Foundation (FMF) preterm preeclampsia (PE) screening algorithm in the South Asian population.	Prospective observational cohort study; A quantitative survey was administered: sociodemographic characteristics, past and current medical history, and pregnancy details.	New Delhi, India	1863 South Asian women carry a singleton pregnancy.	The incidence of preeclampsia and preterm preeclampsia were 3.17% and 1.34% respectively. The FMF algorithm could be further improved to ensure that biochemical markers are adjusted for indigenous South Asian women.	85%
Ramesh et al. (2014) [22]	Socio-Demographic and Other Risk Factors of Pre Eclampsia at a Tertiary Care Hospital, Karnataka: Case Control Study	Determine the sociodemographic and other risk factors of preeclampsia.	Case control study; A quantitative survey was administered that asked for sociodemographic information. Interviews were conducted after.	Karnataka, India	100 cases of preeclampsia and 200 controls from the Vijayanagara Institute of Medical Sciences.	Age of < 20 years, low SES, family history of HDP, family history of diabetes, and family history of hypertension were found to be significant risk factors of preeclampsia.	100%
Raj et al. (2018) [23]	Incidence of Gestational Hypertension Among Pregnant Women in the Rural Population of District Amritsar: A Community Based Study	Determine frequency of hypertension and associated risk factors in the rural community of District Amritsar.	Cross-sectional case control study; Participants were interviewed, received a quantitative survey that included: socio-demographic factors, personal history, family history, general & systemic examination, and obstetric history.	Amritsar, India	189 pregnant Indian women in the Rural Health Centre Mallu-Nangal.	HDPs were identified in 5.8% of the participants. The frequency of HDPs was higher with increasing age, illiteracy, Hindu women, women with > 20 weeks of gestation, past HDPs, and family history of hypertension. Large need for regular prenatal screenings.	85%
Singh et al. (2020) [24]	Identifying the Risk Factors for the Prevention of Hypertensive Disorders in Pregnancy in a Tertiary Care Hospital: A Cross-sectional Study	Identify the demographic profile and risk factors pertaining to lifestyle and behavioral aspects of HDPs.	Cross-sectional study; A quantitative survey was administered that asked for sociodemographic characteristics. Various blood pressure exams were conducted as well.	Mumbai, India	225 pregnant Indian women in a tertiary care hospital in Mumbai.	11.1% of the subjects were illiterate, 50.2% consumed additional salt in their diet, and 25.3% had a history of intake of visible fat. 77.3% did not consume tobacco in any form and 60.9% did not have a family history of hypertension.	100%
Singh et al. (2021) [25]	Effects of Diet on Hypertensive Disorders During Pregnancy: A Cross-Sectional Study From a Teaching Hospital	Understand the role of diet on HDP development.	Cross-sectional study; A quantitative survey was administered that asked for sociodemographic characteristics. Various blood pressure exams were conducted as well.	India	225 hypertensive pregnant Indian women.	The relation between visible fat consumption and HDP was found to be statistically significant. 81.8% of participants were taking mixed diets, 50.2% consumed additional salt in their diet, the intake history of visible fat was given by 25.3% and 96.4% consumed tea while 52.9% had a history of consuming junk food.	100%
Vidler et al. (2016) [26]	Community Perceptions of Preeclampsia in Rural Karnataka State, India: A Qualitative Study	Explore community-based understandings of HDPs.	Qualitative study; Fourteen focus groups were held with community leaders, male decision-makers, female decision-makers, and reproductive age women. Qualitative data was collected.	Karnataka, India	14 focus group discussions with 219 individuals:	‘Pre-eclampsia’ and ‘eclampsia’ are not well-known; hypertension is perceived as conditions that may occur during pregnancy. Improving community knowledge and modifying attitudes towards HDPs and its complications can address delays in disease recognition and treatment.	100%
Khargekar, V. & Khargekar N. (2016) [27]	Clinico-social Profile of Mothers with Pregnancy Induced Hypertension (PIH) Admitted to Hospitals Attached to JJM Medical College, Davangere, Karnataka	Study the sociodemographic profiles of mothers with HDPs to understand different HDP prevalence types.	Longitudinal study; A quantitative survey was administered that asked for sociodemographic characteristics. A clinical examination was also implemented.	Karnataka, India	163 Indian mothers admitted to three hospitals in Karnataka.	51.5% were in age group of 21-25yrs, 74.2% belonged to rural areas, 77.9% housewives, 63.1% lived in joint families and 41% were of low SES.	100%
**Total Quality Score**							94%

See Supplemental Fig. 1 for a map depicting the geographic distribution of included studies.

To showcase the interdisciplinary nature of Bronfenbrenner’s Ecological model, Table 2 organizes the socioecological factors that each study discusses at each level of the model. Studies are mentioned more than once if they are relevant to more than one socioecological factor.

**Table 2:** Socioecological factors associated with HDPs in India extracted from each study, organized within Bronfenbrenner’s ecological model

	Citation	Extracted socioecological factors
**Individual**	Khargekar, V. & Khargekar N. (2016) [27]	Low SES; Low maternal age
	Raj et al. (2018) [23]	High maternal age; Illiteracy; Family and personal history of hypertension
	Padhan et al. (2023) [18]	Family and personal history of hypertension
	Nath et al. (2021) [17]	Low SES; High maternal age
	Singh et al. (2021) [25]	High salt and visible fat diet
	Agrawal & Fledderjohann (2016) [13]	Tobacco-use; Low education level; Family and personal history of hypertension; Low SES
	Singh et al. (2020) [24]	High salt diet; Illiteracy
	Dhinwa et al. (2021) [2]	Low education level; Illiteracy
	Mathew et al. (2023) [15]	High and low maternal age; Family and personal history of hypertension
	Ramesh et al. (2014) [22]	Low maternal age; Low SES; Family and personal history of hypertension
	Grover et al. (2023) [14]	Tobacco-use
	Mehta et al. (2015) [16]	Family and personal history of hypertension; Maternal age
**Microsystem**	Pal et al. (2017) [19]	Lack of community knowledge on diet
	Farrukh et al. (2022) [6]	Lack of community education and knowledge on HDP prevention and management
	Nath et al. (2021) [17]	Low social support
	Vidler et al. (2016) [26]	Lack of community education and knowledge on HDP prevention and management
**Mesosystem**	Dhinwa et al. (2021) [2]	Lack of prenatal visits leads to improper health education
	Grover et al. (2023) [14]	Tobacco-use is higher amongst rural women than urban women
**Exosystem**	Mehta et al. (2015) [16]	Lack of prenatal HDP screening
	Pal et al. (2017) [19]	Lack of prenatal HDP screening
	Prasad et al. (2021) [21]	Lack of prenatal HDP screening
	Mathew et al. (2023) [15]	Lack of prenatal HDP screening
	Agrawal & Fledderjohann (2016) [13]	Lack of public hospital utilization
	Panda et al. (2021) [20]	Lack of prenatal HDP screening
**Macrosystem**	Farrukh et al. (2022) [6]	Culture of adolescent marriages resulting in higher rates of HDPs
	Khargekar, V. & Khargekar N. (2016) [27]	Culture of women being homemakers

### Individual level

The individual level within Bronfenbrenner’s Ecological model aims to understand how individual characteristics and health behaviors contribute to the development of HDPs in Indian women. Major individual risk factors mentioned in 11 of the included papers are: low SES, maternal age, diet, family and personal history of hypertension, low education level, illiteracy, and tobacco-use [2, 13–18, 23–25, 27].

The modifiable factors at this level include: diet, illiteracy, low education level, tobacco-use, and adherence to medication. Singh and colleagues (2021) conducted a study at a prenatal teaching hospital to understand how the diets of preeclampsia patients contributed to their hypertension [25]. They found that 77.1% of the participants who consumed foods high in visible fat developed preeclampsia. This correlation was determined to be statistically significant. Additionally, foods high in sodium, in conjunction with minimal fruit and vegetable intake were considered significant risk factors for HDPs [24]. Even though food drastically varies based on region (i.e., North, North-East, East, Central, Western, and Southern), and is greatly embedded in the culture of community in India, food habits can still be modified with this context in mind [2].

Education level and literacy have also been shown to increase risk of HDPs. In a study by Singh et al., 11.1% of the participants who had HDPs were illiterate [24]. This correlation was determined to be statistically significant. According to Dhinwa and colleagues, Indian women who are illiterate are less likely to visit their local public hospital for prenatal HDP screening because they are unable to communicate their health needs [2]. The authors claim that this results in inadequate prenatal screening, which then results in missed opportunities for diagnosing HDPs. Individuals who are illiterate are also less likely to advocate for their healthcare, ask follow-up questions, and understand their diagnosis [23]. This can lead to inadequate management of their HDPs. Although there are systemic barriers to improving education level and literacy, these risk factors are still theoretically modifiable.

Tobacco use is also a major contributor to the development of HDPs. Grover and colleagues conducted a study that spanned 29 states in India to examine the correlation between tobacco-use and HDPs in pregnant Indian women [14]. They discovered that hypertension during pregnancy was significantly higher amongst tobacco users (7.5%) than non-users (6.1%). The use of tobacco in many forms, like smoking and chewing, is associated with higher rates of HDPs [13]. Even though tobacco-use is heavily dictated by an individual’s environment and socioeconomic pressures, the decision to use tobacco can be modified.

The non-modifiable factors at this level include: low SES, maternal age, and family and personal history of hypertension. In a study, Khargekar, V. and Khargekar N. found that 74.2% of the low SES participants, primarily found in rural India, had HDPs [27]. Similarly, in a study by Nath et al., 15.1% of the hypertensive women in a prenatal clinic in Bengaluru were of low SES [17]. These findings align with those of Ramesh et al. [22]. They found that 80% of the patients with HDPs were earning less than 4000 rupees (~ 48 dollars) a month. Since individuals in rural India are often born into poverty, SES is considered a non-modifiable risk factor.

Being of either low or high maternal age is considered a risk factor for developing HDPs. For mothers older than 35, this is most often due to already developed pre-existing cardiovascular disease risk factors at the onset of pregnancy [28]. For mothers younger than 20, increased risk is due to many factors including uterine immaturity, maternal immune response, potential pre-existing cardiovascular disease risk factors, especially obesity, and socioeconomic factors [28, 29]. One of the most common causes of HDPs in India is being pregnant younger than 20 years of age [15, 22, 27]. In a culture of adolescent marriages, younger pregnancies are more likely, which in turn increases the risk of HDPs [6]. However, being pregnant older than 35 years of age in conjunction with being a first-time mother also elevates the risk for HDPs [15, 17, 23]. Lastly, family and personal history of hypertension serves as a major risk factor for HDPs, which is heavily supported by existing research [13, 15, 16, 18, 22, 23].

### Microsystem level

The microsystem in the socioecological model aims to understand how an individual’s inner environment (i.e., friends, family, neighbors, work, and/or school) influences their health outcomes or behaviors. The major themes in the microsystem determined from 4 studies include: lack of community education and knowledge on hypertension prevention and management; lack of community knowledge on diet; and lack of social support [6, 17, 19, 26].

When asked about what HDPs are, warning signs, and outcomes, individuals in Karnataka, India had major gaps in knowledge [26]. Participants had many perceived causes of preeclampsia, including “short temper,” and “sex of the baby.” The term hypertension was also referred to in many ways, including “BP” [blood pressure], “jhataka” [fits], and “nanju agide” [infection]. The inconsistency in terms and that these terms don’t reflect HDPs indicates the minimal knowledge individuals have on the condition. This can lead to myths about HDPs that may do more harm than good. For example, a female of reproductive age was convinced that, “Those who worry more about their family will have more BP [26].” The shifting perceptions of hypertension and its causes are due to the lack of community knowledge. Regions with home health education on hypertension management showed significant decreases in hypertension cases amongst the adolescent and young adult population [6].

As mentioned at the individual level, many South Asians consume high salt and visibly fat diets (i.e. high fat diets). There is minimal community-based counseling and education on nutrition in India, resulting in the continued consumption of fatty acid rich diets [19]. Additionally, street food is highly prevalent in India due to its convenience and price. It is difficult to measure nutritional value from street food, further contributing to the lack of dietary knowledge regarding food consumption [19].

Lastly, having low social support is a contributor to the development of HDPs. Nath and colleagues examined the socio-demographic variables associated with HDPs. They found that the risk of HDPs was 1.3 times higher in women who reported low social support compared to those who reported high social support [17].

### Mesosystem level

The mesosystem examines how the microsystem and exosystem interact, or how local governments, mass media, and/or extended family influence an individual’s inner circle [7]. For example, an individual’s access to prenatal healthcare can heavily influence their knowledge and perception of HDPs. According to Dhinwa et al., low SES groups, typically located in rural India, are less likely to seek out prenatal clinics, nor meet the minimal recommendation of 4 prenatal visits. As a result, they receive their HDP knowledge and education from community members who are not medical experts [2]. This leads to inaccurate and misguided information on HDP management, causes, and outcomes [26]. This misguided and incomplete education can also explain why tobacco use is higher among rural individuals (8.7%) than urban individuals (6.9%) [14]. Low income, pregnant individuals are not utilizing their local hospitals nor community education clinics, ultimately impacting their health behaviors (i.e., tobacco-use) that increase their risk of HDPs [14].

### Exosystem level

The exosystem considers how local governments, the medical system, and other key infrastructural bodies influence an individual’s health behaviors and outcomes [7]. The major risk factors identified from 6 studies that elevate risk of developing HDPs in this system include: lack of prenatal HDP screening and lack of public hospital utilization [13, 15, 16, 19–21]. Several studies showed that patients who developed HDPs either attended less than 4 prenatal appointments or attended and did not receive adequate HDP screening [15, 16, 19–21]. The prenatal HDP screening that most Indian hospitals use is by the Fetal Medicine Foundation (FMF). Prasad and colleagues conducted an analysis of the efficacy of the FMF screening [21]. They discovered that although the FMF screening performed satisfactorily, it could still be improved to correctly adjust for biomarkers for South Asian women. As a result, the individuals who do attend their prenatal appointments may not receive accurate diagnoses. In addition to the screening being less sensitive, medical providers do not enforce or encourage patients to attend their prenatal appointments [15]. The issue of public hospital utilization contributes to the lack of accurate HDP screening. Women who get their healthcare information from elsewhere, like clinics, NGOs, or friends/family are more likely to have HDPs due to the lack of guidance and implementation of prenatal HDP screening [13].

### Macrosystem level

The macrosystem examines the culture, economic systems, and politics. Farrukh and colleagues evaluated socio-demographic risk factors to understand the high prevalence of HDPs in South Asia [6]. They found that 45% of women with HDPs aged 20–24 years of age reported being married before the age of 18. Being married at a young age can inevitably lead to childbirth at a low age, which as discussed earlier, is a risk factor for the development of HDPs. This culture of adolescent marriages has been shown to increase the risk of HDP development [6]. Additionally, Khargekar, V. and Khargekar N. found that India has a large culture of women being homemakers which contributes to high HDP rates [27]. Being a homemaker is defined as women attending, organizing, and leading the day-to-day responsibilities in the home [27]. In their study, 77.9% of mothers with HDPs were homemakers, a statistically significant correlation.

## Discussion

### Main findings

This rapid review used Bronfenbrenner’s Ecological Model to understand how factors in every system of an Indian birthing person’s life can affect their risk of developing HDPs. Previous research has examined various risk factors of HDPs in India, but this is the first study to use Bronfenbrenner’s ecological model to understand how each of these risk factors interact at a different level of influence. The HDP risk factors that were most commonly identified amongst the 17 studies include: having low SES, consuming a high salt or high fat diet, lacking community education and knowledge on HDP management and prevention, and lacking prenatal HDP screening.

#### Low SES

A common theme amongst most of the included studies is that individuals of low SES (i.e., individuals residing in rural areas) are more likely to develop HDPs [2, 13, 17, 22, 27]. India has a unique distribution of class, largely due to the caste system. Rural or socioeconomically disadvantaged areas are more impoverished compared to urban areas because the caste system actively discriminates and segregates low-income people. When individuals are born into poverty and the rural areas of India, they are subject to deprivation in job opportunities and healthcare resources [30]. Having low SES in India usually means that the individual lacks public hospitals in their community, has low healthcare coverage, and is uneducated about the medical system [31]. As a result, community knowledge on HDPs, prenatal HDP screening, and hospital utilization are low – all important factors in the prevention and detection of HDPs. This can explain why low-income women are more likely to use tobacco, a major risk factor of HDP, and limit the use of public hospitals. They are not receiving adequate health education on lifestyle behaviors and don’t have the ability to screen for HDPs nor communicate healthcare needs to medical professionals. The impacts of having low SES at the individual level spill into the microsystem, mesosystem, and exosystem, illustrating the importance of using a systems approach in understanding HDP development.

Unfortunately, SES is non-modifiable due to the rigid and systemically oppressive nature of the caste system. However, this finding leaves room for many avenues of intervention at both the community (i.e., microsystem) and policy (i.e., macrosystem) levels to bridge the rural-urban gap of health equity. At the community level, requiring HDP education at community clinics or hospitals will help expand rural individuals’ health literacy and knowledge on HDPs. They can also begin understanding what healthy behaviors mean and how to modify them. At the policy level, it would be beneficial to enact clinical guidelines that require at least 4 and ideally 8 prenatal visits to adequately screen for HDPs [2]. This requirement will reduce the burden on an individual who is neither literate nor knowledgeable on HDPs.

#### Consuming a high salt or high fat diet

Diet is closely linked to HDP risk, with high levels of fruits and vegetables, plant-based foods and vegetable oils, and low levels of fat, sugar and salt associated with lower risk for HDPs [32]. In particular in India, high salt and fat diets have been linked at the individual level to risk for HDPs [24, 25]. At the microsystem level, there is an overall lack of community knowledge about the impact of diet on risk for HDPs [19]. There is a trend in India of an increase in availability of highly processed foods, reduced physical activity, and limited access to diverse foods that is leading to an increase in obesity and cardiovascular disease risk factors such as high blood pressure [33, 34]. In particular, a recent study found that regions of India with a higher density of highly processed take-away food were associated with increased blood pressure in comparison to regions with a higher density of fruit and vegetable vendors [35]. This overall shift is contributing to the increased risk for HDPs and is another aspect that could be focused on at the policy level by increasing access to higher quality foods across India. However, it is challenging to change cultural attitudes about dietary choices and would need to be approached in a manner incorporating most of the Bronfenbrenner levels to be effective [36].

#### Lack of community education and knowledge on HDP management and prevention

Lack of community knowledge on HDP management and prevention was associated with higher rates of HDPs [6, 26]. In public health practice, education at the individual, community, and policy levels is encouraged to maximize patient knowledge. This is especially true for diseases like HDPs because prevention and treatment efforts mostly occur at the individual level for modifiable risk factors (e.g., tobacco-use, diet, home blood pressure monitoring). Therefore, if the community fails to educate an individual on HDPs, they are less likely to understand HDP risk, prevention, and management to make lifestyle changes. The impacts of minimal HDP knowledge were displayed in the study by Vidler et al., where participants had inconsistent terms/names when referring to HDPs [26]. Minimal community knowledge is mostly prevalent in rural communities due to systemic barriers in education and literacy. Educational programs should be implemented at the community level for pregnant individuals in rural areas. For example, community clinics could host open seminars or workshops for birthing women to understand HDP development and how it can be prevented or mitigated.

#### Lack of prenatal HDP screening

Lack of prenatal HDP screening in India contributes to the development of HDPs [15, 16, 19–21]. Across multiple studies, common themes included that individuals would attend their prenatal screening appointments, but would receive inadequate treatment from their providers [21] or, they would only attend only one or two appointments, even though 8 prenatal visits are recommended [2]. This insufficient HDP care is out of the individual’s control, yet dramatically impacts their risk of HDP development. This is problematic because HDPs can lead to hypertension and/or CVD in the future creating a cycle of hypertension and CVD in India [5]. Additionally, when healthcare providers fail to encourage or remind their patients to attend prenatal appointments, there are more missed opportunities for HDP diagnosis, especially for individuals who are illiterate or uneducated in rural areas [37]. A possible intervention to increase prenatal visits to catch HDPs early on would be to enact policies that require 4–8 prenatal visits to ensure accurate diagnoses and treatment. It would also be beneficial to increase access to telehealth platforms that remind patients of their appointments.

### Limitations

This rapid review has several limitations. First, interventions and policy recommendations will differ between Indian women in India and other parts of the world. Ultimately, socioecological factors depend on an individual’s environment, so these findings may not be relevant to an Indian woman in a country with drastically different environments than those described in this paper. Next, although this paper followed the PRISMA checklist and employed an extensive search strategy, it was conducted within a short time frame due to the nature of rapid reviews. As a result, the quality appraisal is limited, affecting the findings from this review. Similarly, most of the reviewed papers were quantitative in nature, which prevents examination of rich descriptions that can be obtained from qualitative studies regarding how these factors influence HDPs. Next, all the included studies were written in English, but if the study population did not know English and spoke in their local dialect in interviews, we have no way of knowing if key information was lost in translation. Lastly, many of the studies used in the final review included lower or middle class participants. Therefore, these results cannot be extrapolated to high class individuals due to different standards of living, access to resources, and physiological characteristics.

## Conclusion

Multiple, intertwined socioecological factors influence the development of HDPs among Indian women, the rates of which are high compared to other groups worldwide. To address this inequity, individuals in rural and low SES areas need more health education on HDP management and prevention. There also needs to be more adequate prenatal HDP screening, with at least 4 and ideally 8 prenatal visits [2]. A comprehensive prenatal screening guideline should be developed to ensure that healthcare providers are thoroughly evaluating patients for HDPs. These prenatal screenings should be accompanied with culturally appropriate patient education on the diagnosis, especially for low SES women who have limited literacy, so that they can effectively make individual and microsystemic lifestyle decisions aimed at either managing or preventing HDPs. Lastly, community education to address the risks associated with adolescent marriages are essential in combating HDPs associated with young birthing age. Future research should use Bronfenbrenner’s Ecological Model to explore how familial factors contribute to the high rate of HDPs in India. The studies reviewed in this paper did not analyze the impacts of family culture and relationships, however, we believe that is worth exploring how familial factors influence diet, physical exercise, and prenatal screening habits which ultimately affect HDP risk. In addition, it would be beneficial to look at genetic risk in conjunction with modifiable factors to investigate personalized prevention and treatment options. Finally, more qualitative research in this area could further elucidate how these factors are interacting to influence HDPs.

## Electronic supplementary material

Below is the link to the electronic supplementary material.

Supplementary Material 1

## Data Availability

No datasets were generated or analysed during the current study.

## Abbreviations

BMI: Body Mass Index
CVD: Cardiovascular disease
HDPs: Hypertensive disorders of pregnancy
JBI: Joanna Briggs Institute
MMAT: Mixed Methods Appraisal Tool
SDG: Sustainable Development Goal
SES: Socioeconomic status
