# Supplementary material for: Socioecological factors influencing the risk of developing hypertensive disorders of pregnancy in India: a rapid review
Source: BMC Pregnancy Childbirth. 2024 Oct 12;24:669. doi: 10.1186/s12884-024-06879-0 (PMC11471028; doi:10.1186/s12884-024-06879-0)
Supplement: Supplementary file 1 — Supplementary Material 1 [file 12884_2024_6879_MOESM1_ESM.docx]

**Socioecological factors influencing the risk of developing hypertensive disorders of pregnancy in India: A rapid review**

Anumita Alur, B.S.^1^, Jennifer E. Phipps, Ph.D.,^2^ Leigh Ann Simmons, Ph.D., M.F.T., F.A.A.H.B.^2^

^1^Health Equity Across the Lifespan Lab, University of California, Davis, California, United States

^2^Betty Irene Moore School of Nursing, University of California, Davis, California, United States

**Corresponding author**

Correspondence concerning this paper should be addressed to Jennifer E. Phipps.

Betty Irene Moore School of Nursing, University of California, Davis, 2570 48th St, Sacramento, CA 95817, United States.

Email: [jephipps@ucdavis.edu](mailto:jephipps@ucdavis.edu)

**Supplement**

**Table 1. Boolean search terms for each database**

| **Database** | **Boolean search terms** |
| --- | --- |
| PubMed | ((((((((((((hypertension) OR (high blood pressure)) OR (hypertensive)) OR (preeclampsia)) AND (pregnancy)) OR (pregnant)) OR (birthing people)) AND (Indian)) OR (South Asian)) AND (risk factors)) OR (factors)) OR (socioecological factors)) OR (lifestyle factors) |
| Scopus | hypertension OR high AND blood AND pressure OR hypertensive OR preeclampsia AND pregnancy OR pregnant OR birthing AND people AND indian OR south AND asian AND risk AND factors OR factors OR socioecological AND factors OR lifestyle AND factors |
| Science Direct | ((((((((((((hypertension) OR (high blood pressure)) OR (hypertensive)) OR (preeclampsia)) AND (pregnancy)) OR (pregnant)) OR (birthing people)) AND (Indian)) OR (South Asian)) AND (risk factors)) OR (factors)) OR (socioecological factors)) OR (lifestyle factors) |

**Table 2. Quality assessment for included reviews using the JBI Critical Appraisal Checklist**

|  | Dhinwa et. al. (2021) [2] | Farrukh et. al. (2022) [6] | Pal et. al. (2017) [19] | % |
| --- | --- | --- | --- | --- |
| Is the review question clearly and explicitly stated? | Yes | Yes | Yes | 100% |
| Were the inclusion criteria appropriate for the review question? | Yes | Yes | Yes | 100% |
| Was the search strategy appropriate? | Yes | No | Yes | 66.70% |
| Were the sources used to search for studies adequate? | Yes | Unclear | Unclear | 33.30% |
| Were the criteria for appraising studies appropriate? | Yes | Yes | Yes | 100% |
| Was critical appraisal conducted by two or more reviewers independently? | Yes | Yes | Yes | 100% |
| Were there methods to minimize errors in data extraction? | Yes | Yes | No | 66.70% |
| Were the methods used to combine studies appropriate? | Yes | Yes | Yes | 100% |
| Was the likelihood of publication bias assessed? | Yes | No | No | 33.30% |
| Were recommendations for policy and/or practice supported by the reported data? | Yes | Yes | Yes | 100% |
| Were the specific directives for new research appropriate? | Yes | Yes | Yes | 100% |
| Quality Score | 100% | 72% | 72% | 81% |

**Note**: The JBI checklist uses a scale of Yes, No, Unclear, or Not Applicable. References are from the main manuscript.

**Table 3. Quality assessment for included quantitative and qualitative studies using the MMAT checklist**

| **Qualitative** | Are there clear research questions? | Do the collected data allow to address the research questions? | Does the qualitative approach answer the research question? | Are the qualitative data methods able to address the research question? | Are the findings adequately derived from the data? | Is the interpretation of results sufficiently substantiated by data? | Is there coherence between qualitative data sources & interpretation? | Quality Score |
| --- | --- | --- | --- | --- | --- | --- | --- | --- |
| Vidler et. al. (2016) | Yes | Yes | Yes | Yes | Yes | Yes | Yes | 100% |
| **Quantitative** | Are there clear research questions? | Do the collected data address the research questions? | Is the sampling strategy relevant to the research question? | Is the sample representative of the target population? | Measurements are appropriate? | Is the risk of nonresponse bias low? | Is statistical analysis appropriate to answer research question? | Quality Score |
| Agrawal & Fledderjohann (2016) [13] | Yes | Yes | Yes | Yes | Yes | Yes | Yes | 100% |
| Grover et. al. (2023) [14] | Yes | Yes | Yes | Yes | Yes | Yes | Yes | 100% |
| Mathew et. al. (2023) [15] | Yes | Yes | Yes | Yes | Yes | Yes | Yes | 100% |
| Mehta et. al. (2015) [16] | Yes | Yes | Yes | Yes | Yes | Yes | Yes | 100% |
| Nath et. al. (2021) [17] | Yes | Yes | Yes | Yes | Yes | Yes | Yes | 100% |
| Padhan et. al. (2023) [18] | Yes | Yes | Yes | Yes | Yes | Yes | Yes | 100% |
| Panda et. al. (2021) [20] | Yes | Yes | Yes | Yes | Yes | No | Yes | 85% |
| Prasad et. al. (2021) [21] | Yes | Yes | Yes | Yes | Yes | No | Yes | 85% |
| Ramesh et. al. (2014) [22] | Yes | Yes | Yes | Yes | Yes | Yes | Yes | 100% |
| Raj et. al. (2018) [23] | Yes | Yes | Yes | Yes | Yes | No | Yes | 85% |
| Singh et. al. (2020) [24] | Yes | Yes | Yes | Yes | Yes | Yes | Yes | 100% |
| Singh et. al. (2021) [25] | Yes | Yes | Yes | Yes | Yes | Yes | Yes | 100% |
| Khargekar, V. & Khargekar N. (2016) [27] | Yes | Yes | Yes | Yes | Yes | Yes | Yes | 100% |
| **%** | 100% | 100% | 100% | 100% | 100% | 78.50% | 100% | 97% |

**Note**: The MMAT checklist uses a scale of Yes, No, or Unsure. References are from the main manuscript.

**Supplemental Figure 1. Geographic distribution of included studies**

**
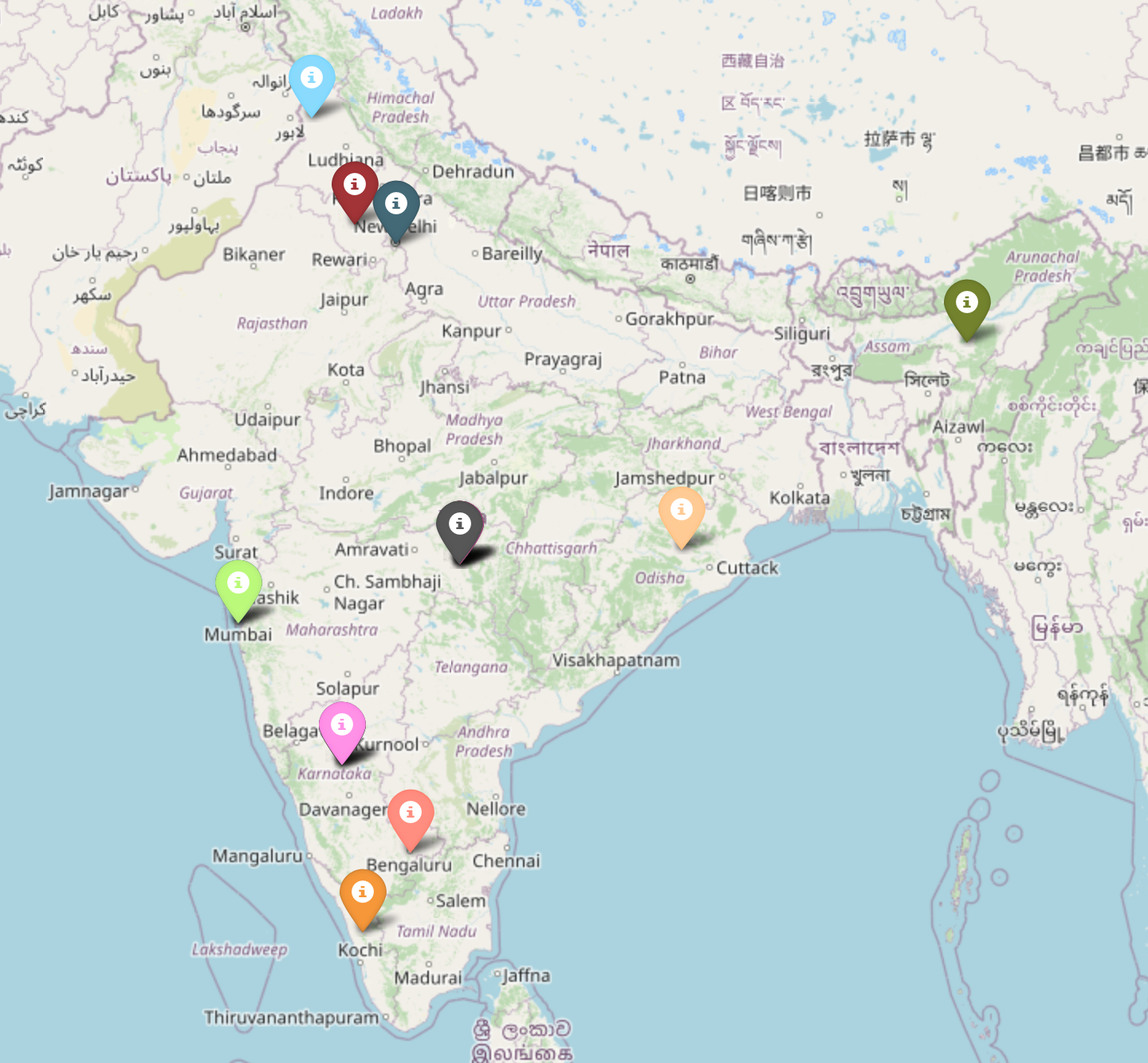
**

**Note**: Blue: Raj et. al. (2018); Red: Mehta et. al. (2015); Teal: Prasad et. al. (2021); Green: Panda et. al. (2021); Yellow: Padhan et. al. (2023); Light green: Pal et. al. (2017), Singh et. al. (2020); Pink: Vidler et. al. (2016), Khargekar, V. & Khargekar N. (2016); Light orange: Nath et. al. (2021); Orange: Mathew et. al. (2023); Dark gray: Agrawal & Fledderjohann (2016), Dhinwa et. al. (2021), Farrukh et. al. (2022), Grover et. al. (2023), Singh et. al. (2021).
